# Supplementary material for: ASD mutations in the ciliary gene CEP41 impact development of projection neurons and interneurons in a human cortical organoid model
Source: Mol Psychiatry. 2026 May 4;31(9):5265–78. doi: 10.1038/s41380-026-03615-2 (PMC13441880; doi:10.1038/s41380-026-03615-2)
Supplement: Supplementary file 1 [file 41380_2026_3615_MOESM1_ESM.docx]

­

**Supplementary Figure 1: *CEP41* mutagenesis and CEP41 protein expression in cortical organoids**. (A) Schematic domain structure of the CEP41 protein with indication of missense mutations found in ASD individuals. (B) Sequencing traces confirming successful mutagenesis. In addition to the R242H mutation, a second silent mutation was introduced to inactivate the PAM site. (C) Schematic of the protocol used to generate cortical organoids. (D) CEP41 Western with protein extracts from D38 control, heterozygous and homozygous mutant organoids. (E) Quantification of the CEP41 Western blot. (F, G) CEP41 expression in cilia of control (F) and in *CEP41*^R242H/R242H^ radial glial cells (G). CEP41 protein was confined to the basal body with lower expression in the axoneme in both genotypes. (H, I) CEP41 protein is absent from the mitotic spindle of radial glial cells as indicated by double staining for γTubulin (arrows). Statistical data are presented as means ± 95% confidence intervals (CI); one-way ANOVA followed by Tukey’s multiple comparisons test with n=3 for all genotypes (E). * p < 0.05. Scale bar: 2.5 μm.

**Supplementary Figure 2: Expression of pluripotency markers in control, *CEP41*^R242H/+^ and *CEP41*^R242H/R242H^ iPSC lines**. (A-I) Immunofluorescence stainings for the indicated markers. All iPSC lines were positive for NANOG (A, D, G), OCT4 (B, E, H), and SOX2 (C, F, I). (J) Schematic diagram depicting the percentage of cells positive for the indicated stem cell markers. Scale bar: 50 μm.

**Supplementary Figure 3: Orientation of the mitotic spindle is not altered in *CEP41* mutant apical radial glial cells**. (A-C) Immunofluorescence staining against pHH3 and pVIM to reveal mitotic cells and the orientation of the mitotic spindle, respectively. The white arrow indicates a horizontal, the purple arrow a vertical and the red arrow an oblique cell division plane (C). (D) Quantification of horizontal (n=238 and 319 mitoses for control and homozygous mutant organoids, respectively, derived from three different lines for each genotype), vertical (n=469 and 647 mitoses for control and homozygous mutant organoids, respectively, derived from three different lines for each genotype) and oblique (n=214 and 257 mitoses for control and homozygous mutant organoids, respectively, derived from three different lines for each genotype) cell divisions. Chi-square test; p=0.9374. Scale bars: 200μm.

**Supplementary** **Figure 4: RG+EN cell type is primarily present in *CEP41* mutant organoids**. (A) UMAP showing the distribution of RG+EN cells over several cell groups. (B) Violin plot revealing the expression of RG+EN characteristic markers. These cells are mainly distinguished by their high *LRRC75A* expression levels and intermediate expression of the histone markers *HIST1H3B*, *HIST1H3D* and *HIST1H2AG*.

**Supplementary Figure 5: Contribution of the three control and three D38 *CEP41* mutant scRNA-seq sample to the various cell clusters**.

**Supplementary Figure 6: Proportions of cortical cell clusters in D38 *CEP41*^R242H/R242H^ mutant organoids**. Bar plots showing the proportions of cortical progenitors to excitatory neurons (A), of RGC subtypes (B), of RGCs in S, G2/M and G1 phases of the cell cycle (C) and of excitatory neuron subclusters (D). Statistical analyses was performed using the propeller() function from the speckle package.

**Supplementary Figure 7: Number of differentially expressed genes per cell cluster in D38 (A) and D94 (B) organoids.**

**Supplementary Figure 8: Contribution of the three control and three D94 *CEP41* mutant scRNA-seq sample to the various cell clusters**.

**Supplementary Figure 9: Proportions of dorsal telencephalic cell types in D94 organoids**. Bar plots showing altered cell-type proportions: dorsal progenitors (aRG and oRG combined) vs excitatory neurons (all ENs combined) (A), apical vs outer radial glial cells (B), upper (EN(UL1-3) vs deep (EN(DL)+EN(LIV-V)+EN(mix)) layer cortical neurons (C) and individual excitatory neuron subclusters (D). Statistical analyses was performed using the propeller() function from the speckle package.

**Supplementary Figure 10: Increased PTN signalling in *CEP41*^R242H/R242H^ mutant organoids at D94**. (A) Heatmap of PTN signal strength in each cell population in control and homozygous mutant organoids.(B) Violin plot showing the expression of *PTN* and its receptors in the D94 cell groups. Statistically significant *PTN* up-regulation as marked by the asterisks is observed in aRG, oRGs, EN(DL) and VP cell populations.

**Supplementary Figure 11: Ventral progenitor and interneuron proportions and interneuron identity in D38 and D94 organoids**. (A-C) Bar plots revealing the proportions of D38 ventral progenitors and interneurons. (D) UMAP indicating the contribution of control (blue) and mutant (red) cell to each cell type at D94. Ventral cell types are highlighted in grey. (E, F) Bar plots showing unaltered proportions of D94 progenitors and interneurons (E) and no change in the proportions of D94 interneuron subtypes(F). (G-L) UMAPs revealing the identity of organoid interneuron populations. Statistical analyses was performed using the propeller() function from the speckle package.

**Supplementary Figure 12**: Violin plots indicating the expression levels of ganglionic eminence markers across the ventral cell types.

**Supplementary Figure 13:** **Differential gene expression in ventral cell populations of D38 organoids**. (A-D) Gene ontology (GO) analysis of up-regulated (left half) and down-regulated (right half) genes in VP1 (A), VP2 (B), IN1 (C) and IN2 (D) clusters. GO terms are ordered by increasing *p*-value from top to bottom; *p*-values are shown colour coded. Count indicates the number of genes associated with each GO term.

**Supplementary Figure 14: Reduced NR2F2 protein levels in D38 *CEP41* mutant cortical organoids.** (A) Quantification of NR2F2 protein levels. (B) Frequency distribution of NR2F2 protein expression. Mann-Whitney test; **** <0.0001

**Supplementary Figure 15:** **Analysis of gene expression changes in D94 ventral cell types**. (A, B) Gene ontology (GO) terms enriched in up-regulated (A) and down-regulated (B) differentially expressed genes (DEGs) in D94 ventral progenitors. Least redundant out of 10 most-significant GO terms shown, ordered by increasing *p*-value from top to bottom; *p*-values are shown inside/next to bars. Count indicates the number of genes associated with each GO term. (C, D) Network analysis showing DEGs associated with the GO terms. Only significant DEGs and GO terms (adjusted *p*-value<0.05) were considered.

**Supplementary Figure 16: Activity of the SHH signalling pathway in *CEP41*^R242H/R242H^ mutant organoids**. (A, B) Dot plots showing the average (colour) and percentage (dot size) expression of housekeeping genes and SHH signalling markers across ventral cell types in D38 (A) and D94 (B) cortical organoids. Note the low percentage expression of most analysed markers. (C) qRT-PCR analyses showing *GLI1* mRNA expression relative to *ATP5* in D47 ventral organoids. (D, E) Immunofluorescence staining for SMO and ARL13B. (F) Quantification of the SMO/ARL13B intensity ratio. Statistical data are presented as mean ± standard deviation (C) and means ± 95% confidence intervals (F); unpaired t-test (C) and Mann-Whitney test (F). n=3 for control and mutant lines. n = 45 cilia from three different lines for each genotype; * p < 0.05; scale bar in (D): 2.5 μm.

**Supplementary Figure 17: The *CEP41* mutation specifically decreases the proportion of NR2F2+ cells in ventral organoids*.*** Immunostaining analyses using the indicated markers. **(A–H)** DLX2 and OLIG2 expression in control and mutant organoids. (I-L) Quantification of DLX2+ and OLIG2+ cell densities relative to DAPI+ area in organoids (I, J) and lines (K, L). DLX2+ and OLIG2+ cell densities showed no significant changes. (M, N, Q, R) SOX2 and NR2F2 expression. Quantification of NR2F2+ and SOX2+ cell densities in control and mutant organoids (O, S) and lines (P, T). The NR2F2+ but not the SOX2+ cell density was decreased in *CEP41*^R242H/R242H^ organoids. Statistical data presented as mean ± standard deviation (SD); control (n=11-12) and mutant (n=16) organoids; control (n=3) and mutant (n=4) lines; unpaired t tests (J-L, P, S, T); Mann-Whitney test (I, O); **** = *p*<0.0001; *** = *p*<0.001; ns = *p*>0.05.Scale bars, 250 μm (overviews) and 100 μm (magnified images).

**Supplementary Figure 18: GLI3 binds to key genes controlling ventral telencephalic development**. Genome browser snapshots showing GLI3 ChIP-peaks in the intergenic regions of *DLX1/2* (A) and *DLX5/6* (B), and in the promoter regions of *NR2F1* (C) and *NR2F2* (D).

**Supplementary Figure 19:** **NR2F1 binds to genes controlling GABAergic interneuron development**. Genome browser snapshots showing NR2F1 ChIP-peaks in the *NR2F1* promoter (A), in the intergenic regions of *DLX1/2* (B) and *DLX5/6* (C), and in the promoter regions of *LHX6* (D), *GAD1* (E) and *CALB1* (F). In each case, the upper lane shows binding of wild-type NR2F1 protein, while the lower lane represents binding of NR2F1-R112K mutant protein as a negative control.
